# The ultimate question? Evaluating the use of Net Promoter Score in healthcare: A systematic review

**DOI:** 10.1111/hex.13577

**Published:** 2022-08-19

**Authors:** Corey Adams, Ramesh Walpola, Anthony M. Schembri, Reema Harrison

**Affiliations:** ^1^ St Vincent's Health Network Sydney Sydney New South Wales Australia; ^2^ School of Population Health University of New South Wales (UNSW) Sydney New South Wales Australia; ^3^ Faculty of Medicine, Health and Human Sciences, Australian Institute of Health Innovation Macquarie University Sydney New South Wales Australia

**Keywords:** Friends and Family Test, healthcare, improvement, measurement, Net Promoter Score, patient experience, quality

## Abstract

**Background:**

Patient experience is a complex phenomenon that presents challenges for appropriate and effective measurement. With the lack of a standardized measurement approach, efforts have been made to simplify the evaluation and reporting of patient experience by using single‐item measures, such as the Net Promoter Score (NPS). Although NPS is widely used in many countries, there has been little research to validate its effectiveness and value in the healthcare setting. The aim of this study was to systematically evaluate the evidence that is available about the application of NPS in healthcare settings.

**Methods:**

Studies were identified using words and synonyms that relate to NPS, which was applied to five electronic databases: Medline, CINAHL, Proquest, Business Journal Premium, and Scopus. Titles and abstracts between January 2005 and September 2020 were screened for relevance, with the inclusion of quantitative and qualitative studies in the healthcare setting that evaluated the use of NPS to measure patient experience.

**Results:**

Twelve studies met the inclusion criteria. Four studies identified benefits associated with using NPS, such as ease of use, high completion rates and being well‐understood by a range of patients. Three studies questioned the usefulness of the NPS recommendation question in healthcare settings, particularly when respondents are unable to select their service provider. The free‐text comments section, which provides additional detail and contextual cues, was viewed positively by patients and staff in 4 of 12 studies. According to these studies, NPS can be influenced by a wide range of variables, such as age, condition/disease, intervention and cultural variation; therefore, caution should be taken when using NPS for comparisons. Four studies concluded that NPS adds minimal value to healthcare improvement.

**Conclusion:**

The literature suggests that many of the proposed benefits of using NPS are not supported by research. NPS may not be sufficient as a stand‐alone metric and may be better used in conjunction with a larger survey. NPS may be more suited for use in certain healthcare settings, for example, where patients have a choice of provider. Staff attitudes towards the use of NPS for patient surveying are mixed. More research is needed to validate the use of NPS as a primary metric of patient experience.

**Patient or Public Contribution:**

Consumer representatives were provided with the research findings and their feedback was sought about the study. Consumers commented that they found the results to be useful and felt that this study highlighted important considerations when NPS data is used to evaluate patient experience.

## 1 INTRODUCTION

Improving patient experiences in healthcare is increasingly becoming a core strategic imperative and is fundamental to global healthcare performance.^1^ The term ‘patient experience’ refers to ‘the sum of all interactions shaped by an organization's culture that influence patient perceptions across the continuum of care’.^2^ While patient satisfaction and complaint resolution are important, focusing solely on these has limitations, which have become increasingly apparent.^3^, ^4^ A key driver for the increased focus on patient experience is the correlation between experiences of healthcare and the safety and quality of care provision.^5^ A positive patient experience is associated with fewer adverse safety events, more favourable perceptions of safety event handling (including disclosures) and a lower risk of litigation in the aftermath of a safety event.^6^ Due to the strong association with quality and safety in healthcare, enhancing the patient experience is identified as a goal of the ‘Quadruple Aim’ framework, developed by the Institute for Healthcare Improvement^7^ and is used as an indicator for healthcare accreditation with the National Safety and Quality Health Service Standards.^8^ The inclusion of patient experience as an outcome indicator has necessitated the development of measurement tools for capturing, monitoring and benchmarking performance.

While many healthcare organizations strive to improve the patient experience, it has proven difficult to rigorously evaluate. There is no single, best‐practice method to measure patient experience,^9^ and this limits the ability to benchmark patient experience across departments, organizations and countries.^10^ A number of measurement tools have been developed to assess patient experience, such as the multi‐item Hospital Consumer Assessment of Healthcare Providers and Systems (HCAHPS) survey, which is often used in the United States,^11^ and the 12‐item Australian Hospital Patient Experience Question Set (AHPEQS) developed by the Australian Commission on Safety and Quality in Healthcare.^12^

As healthcare organizations regularly report on outcome measures, surveying of patients typically produces a large volume of data and increased demands associated with data management. Therefore, to simplify data management and reporting, healthcare organizations may prefer to use information that is condensed into a single performance indicator, or ‘composite measure’.^13^ Patient experience is a complex and multifaceted phenomenon, however, which can be difficult to condense into a single outcome indicator. One method to achieve this is using the single‐item Net Promoter Score (NPS). Created in 2003, NPS has been used in a variety of industries around the world, including banking, insurance and technology.^14^ In recent years, NPS has been adopted into healthcare settings, frequently for the purpose of system‐level benchmarking.^15^ NPS is a popular surveying method that is used globally and has been dubbed ‘the ultimate question’.^16^

**Figure 1:**
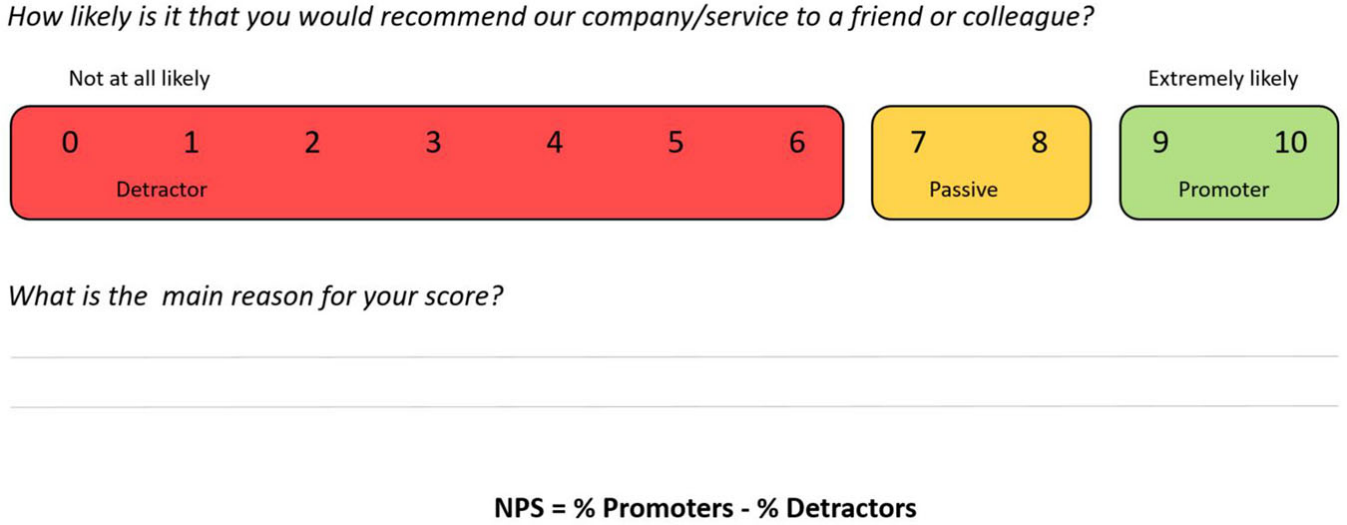
Categorization and calculation of Net Promoter Score (NPS).

NPS consists of a two‐part questionnaire. Firstly, respondents are presented with a rating question: ‘How likely is it that you would recommend our business/service to a friend or colleague?’ on a scale from 0 (*not likely*) to 10 (*very likely*). This is followed by an open‐ended, free‐text item, which enables respondents to provide the main reason for their score. Based on the rating that is provided, responses are categorized into three groups: ‘Detractors’ (ratings 0–6), ‘Passives’ (ratings 7 and 8) and ‘Promoters’ (ratings 9 and 10). The overall score, known as the ‘Net Promoter Score’, is calculated by subtracting the percentage of Detractors from the percentage of Promoters, therefore NPS can range from −100 (i.e., all Detractors) to +100 (i.e., all Promoters; see Figure 1).^16^

**Figure 2:**
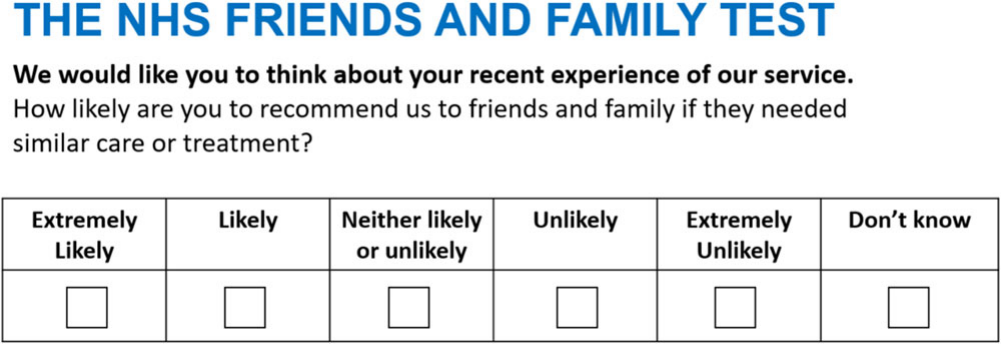
The NHS Friends and Family Test. NHS, National Health Service.

NPS is widely used in healthcare settings internationally. For instance, in 2012, the UK National Health Service (NHS) implemented a modified version of the NPS survey, called the ‘Friends and Family Test’ (FFT), which became a nationally mandated measure to monitor consumer satisfaction and healthcare quality performance.^9^ This replaced the NPS numerical rating (i.e., 0–10) with a 5‐point Likert scale to measure willingness to recommend health services (which ranged from ‘Extremely Likely’ to ‘Extremely Unlikely’; see Figure 2). For improved comprehension, FFT will be categorized as NPS in this review.

NPS may be appealing as a simple and quick single‐item measure to evaluate patient experience performance, however there is a shortage of evidence regarding the suitability of the NPS to assess patient experience in healthcare settings and the limitations of its use.^16^ As a result, this systematic review aims to address this evidence gap to systematically evaluate the application of NPS in healthcare settings to measure Patient Experience. We sought to address the following review question: What evaluative evidence is available about the application of NPS in healthcare settings?

## 2 METHODS

This systematic review was reported in accordance with the Preferred Reporting Items for Systematic Review and Meta‐Analyses statement.^17^

### 2.1 Inclusion criteria

Studies were eligible for inclusion that were published in peer‐reviewed journals since 2005, included the use of NPS in healthcare settings and were available in English. Studies were eligible that included patients of any age or healthcare setting in which the NPS was used for measurement of patient experience, and in which the study reported evaluative data on the use of NPS. This included studies of any research design using quantitative, qualitative and mixed or multimethods.

### 2.2 Exclusion criteria

Studies were excluded if the NPS was used as an outcome measure within a research study and there was no evaluative data about the use of the NPS. Due to the focus on evaluation of peer‐reviewed research, other forms of publications (such as case studies, commentaries, and editorial pieces) were not eligible for inclusion.

### 2.3 Study identification

The study identification was conducted in consultation with a medical librarian. A range of relevant text words, synonyms and subject headings for the topic of NPS and healthcare were generated. The search terms used were ‘Net Promoter*’ OR ‘Friends and Family Test’ AND ‘Health’. The search terms were applied to five electronic databases: Medline, CINAHL, ProQuest, Scopus and Business Journal Premium in May 2021. A preliminary search indicated a high volume of literature from the United Kingdom. Therefore, we carefully reviewed the search strategy and terminology to ensure that it was reflective of the use of NPS in other locations before proceeding to complete the search study identification process. Results were merged using reference management software, Endnote (version X9.2; Clarivate Analytics) and duplicate journal articles were removed using Covidence systematic review software (Veritas Health Innovation).

### 2.4 Study selection and data extraction

One reviewer (C. A.) conducted initial title and abstract screening using Covidence. Studies that appeared eligible for inclusion were subject to full‐text screening. Full‐text copies of the publications were obtained and uploaded to Covidence systematic review software. Two reviewers (C. A. and R. W.) independently applied the inclusion and exclusion criteria. Disagreements were discussed and resolved through mutual agreement with a third reviewer (R. H.). The following data were extracted: author, year, location, methodology, study objectives and key results regarding the review objectives.

### 2.5 Assessment of study quality

Studies were critically appraised using the 16‐item Quality Appraisal for Diverse Studies (QuADS) tool, which has demonstrated good reliability and validity to evaluate multiple types of study designs.^18^ C. A. conducted the quality review for all included studies, and these were re‐assessed by reviewers (R. H. and R. W.), with ratings compared to determine the agreement between the two reviewers. Inter‐rater reliability was calculated using the *κ* test and 67.3% agreement was achieved, which indicates substantial agreement for quality appraisal.

### 2.6 Data synthesis

Due to the range of studies included, a narrative synthesis was performed to combine the findings from the different studies. This involved identifying the key findings from each study, and a summary of these findings was synthesized into a narrative text form. Coding occurred with input from three researchers (C. A., R. H. and R. W.), and regular meetings were held (fortnightly) to discuss and reconcile any disagreements, which produced the final thematic categorization.

### 2.7 Excluded studies

Studies were excluded after full‐text review (*n* = 64) because studies were the wrong study design, such as editorials (*n* = 52), wrong comparator, such as did not evaluate NPS (*n* = 10), or were not conducted in a healthcare setting (*n* = 2).

Stakeholders were consulted about the preliminary review findings to support the analytical process. In this study, consumer representatives were provided with the preliminary findings and feedback was sought about the approach to categorizing the research data. Their feedback was used to finalize the themes and categories that were used in the paper. Consumer representatives highlighted the importance of the findings, including relevancy to patient experience measurement and healthcare system improvement.

## 3 RESULTS

**Figure 3:**
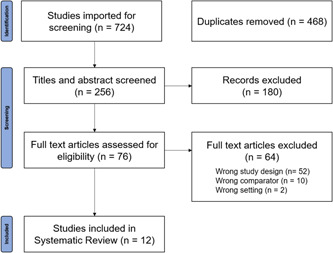
PRISMA flow diagram for the study selection process. PRISMA, Preferred Reporting Items for Systematic Review and Meta‐Analyses.

A total of 468 studies were obtained from the database search. Duplicates were removed, and 256 studies were subject to title and abstract screening. A total of 180 studies were excluded in the first stage of screening, most commonly because the studies were commentary articles. As a result, 76 articles were subject to full‐text screening, from which 64 studies were excluded, most commonly because the studies did not evaluate the use of NPS. Finally, 12 articles were included in the systematic review (see Table 1). The search and selection process is outlined in Figure 3.

**Table 1:** Summary of included studies

Author/s	Year	Location	Setting	Participants	Method	Objectives	Relevant findings
Busby, Matthews, Burke, Mullins and Schumaker	2015	UK	64 Dental practices (64)	10,810 Patients	Surveys	To investigate the relationship between perceived quality and patients' tendencies to recommend a practice to friends and colleagues.	NPS may provide broad but valuable insights into patient perceptions. Benchmarking is most useful within a particular sector.
Gerrard, Jones and Hierons	2017	UK	Primary care oral practices (3)	2019 Patients (Round 1) and 202 patients (Round 2)	Surveys	To test the suitability of PREMs and PROMs surveying.	Staff reported that NPS surveying was time‐consuming and resource‐intensive. NPS (FFT) is subject to various biases. Data should be used for local analysis and improvement, rather than as a comparison between providers.
Hamilton, Lane, Gaston, Patton, MacDonald, Simpson and Howie	2014	UK	Orthopaedic centre (1)	6186 Patients	Surveys	To quantify NPS for joint replacement and ascertain how care is benchmarked against nonhealthcare services, and assess which factors influenced the patients' recommendation response.	There were high rates of completion for recommendation questions. Case mix should be taken into account when comparing scores between hospitals and departments. NPS differences between procedures suggest no overarching score should be given without case‐mix adjustment.
Koladycz, Fernandez, Gray and Marriott	2018	India, Kenya, Nigeria and El Salvador	Family planning clinics	188 Patients in Mumbai, 590 patients (in nine clinics) in Kenya and Nigeria, and 226 patients (in three clinics) in El Salvador	Surveys and interviews (face‐to‐face and telephone)	To test the feasibility and acceptability of implementation approaches in low‐resource clinical settings; to assess whether the methodology could be used to generate meaningful comparative information about the experience of different client groups; and assess the feasibility of a self‐administered NPS using tablets.	Clients (in India) understood the NPS question without additional explanation. Participants may feel challenged to offer suggestions for improvement. Targeted feedback may be necessary to identify actions for improvement, including asking specific questions. Staff reported that NPS was easy to complete. NPS can be used effectively in low‐resource settings with low literacy.
Krol, Maarten W.; Boer, Dolf; Delnoij, Diana M.; Rademakers, Jany J. D. J. M.	2015	The Netherlands	Acute hospitals (6)	6018 Inpatient surveys	Surveys	To assess what the NPS adds to patient experience surveys, and to establish whether NPS is a valid measure for summarizing patient experiences.	Patient experience had a weaker association with NPS than global rating and overall score. NPS is less valid as a summary of patient experience than a global rating. Patients thought it was unusual to recommend healthcare. NPS may oversimplify results, so should be used as an addition to the survey results.
Lawton, O'Hara, Sheard, Reynolds, Cocks, Armitage and Wright	2015	UK	Hospital wards (33)	822 Patients and 648 staff	Surveys	To investigate whether the safety information provided by patients is different from that provided by staff, and whether it is related to safety outcomes.	NPS was associated with patient perceptions of safety, but not associated with safety outcomes. Patient Measure of Safety may be a better indicator of patient safety than NPS.
Manacorda, Erens, Black and Mays	2017	UK	General practices (40)	118 Staff, with clinicians, practice managers and patient representatives	Semi‐structured interviews	To examine the views of practice staff and patients of NPS (FFT), how results are used and to recommend improvements.	NPS questions may be inappropriate (e.g., due to limited choice for GP, and lack of detail in answers to explain reasons). 4 of 42 GP clinics expressed positive views about the use of NPS; only one clinic provided an example of how results had improved quality. NPS is easy to implement, but data had minimal impact on quality improvement. Suggest removing NPS questions and providing a more targeted, specific survey.
Marsh, Peacock, Sheard, Hughes and Lawton	2018	UK	Hospitals	Database search	Scoping review	Review and categorize the types of patient experience feedback data available to understand their function in quality improvement.	Hospitals are challenged to manage the volume of mandated feedback. Mandated patient experience data (including FFT) offers little ready‐to‐use data for quality improvement.
Medforth and Rooksby	2017	UK	General and dental practices (8)	Children	Survey review and case study	To use quantitative data from the Children and Young Person Friendly Friends and Family Test (CYPFF) to assess the impact that the CYPFFT resources have on the uptake of the FFT by children and young people.	NPS was perceived as simple and can be used by children with special needs. NPS survey was completed by a wide range of ethnicities. NPS survey is useful but may add an administrative burden. NPS may raise awareness about the importance of feedback.
Sizmur, Graham and Walsh	2015	UK	Hospitals (32)	38,998 Inpatients and 29,610 ED attendees	Secondary analysis of data collected	To investigate the impact of demographic factors and the mode of administration of a national patient experience questionnaire (FFT).	NPS (FFT) is vulnerable to bias from demographic factors and mode of administration.
Stirling, Jenkins, Clement, Duckworth and McEachan	2019	UK	Centre for hand surgery (1)	810 Patients	Surveys	To quantify NPS for specific hand surgery, identify contributing factors and evaluate the suitability of NPS as an outcome measure for hand surgery.	NPS varied from 44 to 83 for different hand surgeries, which provides a comparison between surgery types. NPS is a useful addition to support the evaluation of clinical value.
Wilberforce, Poll, Langham, Worden and Challis	2018	UK	Mental health teams for older people	352 Respondents	Surveys	To explore the value of NPS as a service improvement tool and outcome measure.	NPS was negatively related to age. NPS may produce value if part of wider data collection, and with a larger sample of service users. Caution is needed when comparing NPS from patients with different prognoses. It may be better to develop a quality score from multi‐item measures, which would provide more breadth and improved reliability.

Abbreviations: FFT, Friends and Family Test; GP, general practitioner; NPS, Net Promoter Score; PREM, patient‐reported experience measure; PROM, patient‐reported outcome measure.

### 3.1 Characteristics of included studies

Ten out of twelve studies were conducted in the United Kingdom, which may reflect the national adoption of NPS measurement (via FFT). One study was conducted in the Netherlands, and NPS was also researched in developing countries (Mumbai, Kenya, and Nigeria). Although NPS originated in the United States, there was a lack of US‐based research. Research was conducted in a range of healthcare settings, including acute hospitals (5), dental practices (3), general practitioner (GP) clinics (2), orthopaedic centres (2), family planning centres (1) and community mental health services (1). One study was conducted in both general and dental practices. The majority of studies examined the use of NPS to evaluate adult patient experience (*n* = 11), while one study focused on paediatric patient experience. The majority of the studies (9) used a survey format, two studies used interviews and one study was a scoping review.

### 3.2 Study quality

Assessment of study quality using the QuADS tool demonstrated variation in the quality assessment criteria for the included studies. The highest scoring areas for study quality included: statement of research aims and objectives, description of data collection procedure and appropriateness of study design to address the research aims. Conversely, lower scoring areas of study quality included: theoretical underpinning to the research and justification for the analytical method selected. In addition, the majority of studies (8 out of 12) received a nil score for evidence that the research stakeholders have been considered in the research design of conduct, which highlights the need for better consumer and stakeholder engagement with research codesign.

The narrative synthesis identified three categories in accordance with the research objectives: context and factors for NPS use, application of NPS for service improvement and considerations for implementing NPS (and managing NPS data) in the healthcare context.

### 3.3 Evaluative evidence of NPS in healthcare

From the 12 studies reviewed, four studies identified that there were benefits associated with using NPS to evaluate patient experience.^14^, ^19^, ^20^, ^21^ Research indicated that NPS can be easily used by a wide range of patients, including adults with low health literacy^19^ and children.^20^ Furthermore, NPS surveying has shown high completion rates by patients, with the ability to generate a large volume of data.^14^ Conversely, 3 of the 12 studies questioned whether the NPS recommendation question was appropriate in the healthcare setting, particularly if consumers have limited choice of healthcare providers.^16^, ^21^, ^22^ For instance, in a study of GP clinics, patients were frequently confused by the recommendation question, particularly because they had limited or no option to choose their GP.^22^

This review identified that the most useful component of NPS surveying was the patient comments section, which was noted to be beneficial in 4 out of 12 studies.^19^, ^21^, ^23^, ^24^ The comments section in NPS is well‐used by patients, typically completed by more than three‐quarters of respondents,^23^ and these comments may help to contextualize the quantitative results gathered from NPS surveying.^25^ For that reason, following a review of the FFT (in 2014), the open‐ended question became mandatory due to the perceived value of these comments.^22^ Alternatively, some healthcare practitioners have reported insufficient detail in the patient NPS responses, which may lack specific information about the causes of patient dissatisfaction^22^ and/or constructive comments about how to improve healthcare services.^19^

### 3.4 Applications of NPS for monitoring and benchmarking

Although NPS has often been implemented for the purpose of monitoring and benchmarking performance, researchers have raised concerns about the validity of NPS data when used to compare healthcare services. Three studies found that NPS results can be influenced by a variety of system and service factors^14^, ^21^, ^24^ which suggests that NPS may be indicative of a wider range of system and service features apart from patient experience. Two studies found significant variation in NPS based on condition and intervention, such as mental health^21^ and type of joint replacement surgery.^14^ For example, one study identified significant variations in NPS between procedures, with NPS of total hip replacement as 71, while NPS of total knee replacement was 49.^14^ As such, caution may be required when comparing NPS between prognoses and conditions.^21^ In addition, NPS may be influenced by various factors, such as mode of administration, gender and patient age.^24^ For instance, two studies reported that patients over the age of 70 had a lower willingness to recommend services.^21^, ^24^ When analysing NPS data, therefore, differences between demographic groups may also need to be considered. NPS may be frequently promoted as a comparison and benchmarking tool, yet researchers have recommended that comparisons of results should be localized.^24^, ^26^ For example, the comparison of NPS results may be restricted to a single hospital site.^24^ Additionally, two studies noted that NPS can be influenced by differences in cultural norms and expectations, which may limit the ability to compare and benchmark NPS data internationally.^16^, ^19^ For example, a score of 8 may be considered to be very good in some cultures, but is classified as neutral (‘Passive’) in NPS methodology.^16^ Cultural considerations regarding NPS responses may impact both sections of NPS, influencing the rating and comments sections. For example, a study about NPS use in India revealed that clients were hesitant to provide critical and constructive feedback about how to improve healthcare services, possibly due to a deferential culture and reluctance to provide critical commentary in the survey.^19^

### 3.5 Evidence for NPS to drive improvements in healthcare

Despite the widespread implementation, there is little evidence of NPS being used successfully for healthcare improvement. Four studies suggested that NPS provides a large volume of information; however, this has limited value to support meaningful healthcare improvement.^21^, ^22^, ^25^, ^27^ For instance, in one study of GP practices, only 1 in 42 clinics gave an example of how NPS results had led to quality improvement.^22^ For this reason, researchers have proposed that more specific questions are required, in addition to NPS, to collect actionable insights that will support service improvement.^22^

NPS has been compared to other methods of evaluation, such as global rating, which requires patients to provide an overall rating of their care using the question: ‘How would you rate the hospital/clinic?’ from 0 (‘worse possible hospital’) to 10 (‘best possible hospital’). Researchers concluded that global rating had stronger associations with quality indicators and patient experience (as measured by the Consumer Quality Index survey) than NPS.^16^ In addition, researchers have also concluded that NPS may not support safety outcome prediction, and there are more accurate measures of proactive patient safety, including the patient measure of safety (PMOS).^27^

### 3.6 Is NPS alone sufficient to measure patient experience?

The benefits of NPS may relate to the perceived simplicity of data collection. However, four studies concluded that NPS alone may be an insufficient measure,^14^, ^19^, ^21^, ^28^ noting that composite measures can oversimplify results and should be used with caution.^13^ Despite the lack of specificity, this information may still provide broad but useful insights.^26^ One study recommended that summary scores should be only used to supplement the results of a larger survey set^16^ and three studies suggested that NPS may be better suited as part of the larger feedback process.^14^, ^19^, ^28^ Researchers have proposed that multi‐item instruments may be more useful than NPS to provide a greater breadth of evaluation and improved reliability.^21^ For these reasons, rather than being a singular encompassing metric, NPS may be better utilized as a starting point to better understand patient experience, which can help to identify areas that require further investigation and detailed examination.^19^

### 3.7 Patient and staff attitudes towards NPS

Two studies noted that the NPS question was well‐understood by a diverse range of patients, including those with low literacy.^19^, ^20^ However patients may also experience concerns about the recommendation question, particularly if they do not have a choice of healthcare provider.^22^ While patient attitudes are quite positive, there are mixed results about staff attitudes towards NPS (from both managerial and clinical staff). One study reported positive staff attitudes towards NPS, which was perceived as being quicker and easier to implement than existing survey methods,^19^ yet this contrasts with the results of two studies that reported negative employee attitudes towards NPS.^22^, ^23^ In one study, staff in dental practices reported NPS to be time‐consuming and resource intensive to implement and maintain,^23^ while in a study of 42 GP practices, only 10% of GP practices expressed positive attitudes towards NPS,^22^ with staff reporting that NPS provided minimal useful information in comparison to existing survey methods.

## 4 DISCUSSION

When measuring patient experience, methods for evaluation need to be capable to assess the complexity of healthcare delivery, and also suitable to the particular healthcare setting.^10^ Although NPS has been used in a wide range of service industries, it has not been validated for use in the healthcare setting. The aim of this systematic review was to evaluate the suitability of the NPS to assess patient experience and to identify potential limitations of its use in the healthcare setting. Healthcare organizations may seek to maximize patient experience, but there is currently no best‐practice measurement tool to measure patient experience.^9^ Consequently, healthcare organizations may adopt tools used in other service industries to measure customer experience, such as NPS. NPS is used in healthcare organizations around the world and has been implemented in a range of healthcare settings, including acute care, primary care and community services. Although NPS has contributed to a large collection of patient feedback, this review highlights that there is a lack of published research to examine the findings about NPS use in healthcare, and limited research to evaluate subsequent improvements derived from NPS surveying.^21^

From a review of 12 eligible studies, it was identified that there are mixed results regarding the usefulness of NPS data in healthcare. Our findings suggest that NPS has immediate benefits, such as being understood by a wide range of patients^19^, ^20^, ^21^ and eliciting a large number of responses.^14^ Furthermore, NPS could provide several indirect benefits, such as encouraging staff and patients to focus on the importance of patient experience and feedback, as well as encouraging the use of real‐time patient surveying. However, staff attitudes towards NPS are mixed. While some staff praised the ease of collecting data via NPS,^19^ others have criticized the lack of useful information generated and expressed concerns about the resources required to manage and maintain the amount of NPS data obtained.^22^, ^23^ This review also finds that NPS may be more appropriate for use in particular healthcare settings. The NPS recommendation question may be confusing for some patients, particularly if they do not have the option of selecting their healthcare provider.^22^ For this reason, NPS may be better suited to elective healthcare settings where the consumer has the ability to choose healthcare providers,^22^ such as dental clinics, physiotherapists and general practitioners.

NPS is frequently used to support benchmarking and comparison of healthcare performance, such as the mandated use of the FFT in the United Kingdom;  however, a review of the research indicates that benchmarking using NPS may be problematic. For instance, researchers have determined that NPS may be influenced by extraneous factors, such as medical condition and respondent age.^14^, ^21^, ^24^ Furthermore, NPS ratings may also be affected by cultural considerations, including cultural differences in rating norms,^16^, ^19^ which may have an impact on the ability to compare NPS results internationally. As such, NPS may be more useful when monitoring longitudinal performance (such as change over time) for a more specific group, such as one hospital site, department and/or patient cohort.^24^ NPS may also be better used to evaluate discrete cohorts, such as categorized according to admission type (i.e., emergency or elective admission), with localized and site‐specific NPS evaluation.

Finally, although NPS is often implemented to support healthcare improvements, this review finds that NPS has limited ability to support healthcare improvement.^13^ While NPS can generate a large volume of data, due to its high completion rates and ease of use,^14^ a review finds that this does not necessarily translate to successful healthcare improvements.^16^, ^21^, ^22^, ^25^ NPS data may be too simplistic and lacks constructive and actionable insights.^14^, ^19^, ^21^, ^29^ Although patient comments can provide useful contextual information, they may also lack sufficient detail and insights to guide healthcare improvement projects.^21^, ^25^ In addition, although NPS is often promoted as ‘the ultimate question’, our research indicates that NPS is not comprehensive enough to be used as a stand‐alone measure for healthcare monitoring. This finding has been supported by research conducted into the use of NPS in other sectors, such as marketing, which concluded that the NPS single‐item question was outperformed by multimetric evaluations.^31^, ^32^ Rather than relying upon NPS as a single guiding metric, NPS may be best suited to provide a high‐level overview of patient experience, helping to identify areas that require further investigation. Alternatively, NPS may be incorporated as part of a larger survey set, with the addition of more comprehensive and targeted questions.^14^, ^19^, ^28^ In doing so, NPS could deliver more value when used in conjunction with a larger feedback process.

This review has identified that there is limited usefulness for NPS in healthcare. Some of the proposed benefits that encourage the use of NPS to evaluate patient experience, such as benchmarking capabilities, are not supported by the existing research. Similarly, research conducted in business marketing concluded that many of the claims initially made to promote the use of NPS, such as the ability to predict customer loyalty, were not supported (or validated) by subsequent research and analysis.^32^ As such, the rigour of using NPS as a tool for patient experience measurement, including validity and reliability of NPS, remains uncertain.

### 4.1 Implications for policy, practice and research

This review highlights that there is a lack of evaluative evidence regarding the effectiveness of NPS to capture the patient experience and improve healthcare services. However, this supports more realistic scoping and utilization of NPS and outlines circumstances in which NPS may be more appropriate for use. There are several important factors to consider with regard to the use of NPS in healthcare. Firstly, it is important to consider the setting. NPS may be more appropriate in specific healthcare contexts, such as elective treatments. Also, because patient ratings and comments can be influenced by cultural factors, there are cultural considerations to be made. While NPS is relatively quick and easy to implement and is well‐completed by patients, healthcare organizations may require additional effort and resources to successfully implement NPS, particularly for staff‐related demands, such as staff training and engagement.

In clinical practice, it is important to understand the context and causality underpinning the metrics, rather than focussing on the NPS number. Ultimately, research suggests that there may be better methods of surveying patient experience than NPS, such as overall rating. Accordingly, NHS recently changed the FFT questions, transitioning from NPS recommendation to overall rating.^29^ This method may be equally as simple to implement as NPS, but it may also be more intuitively understood by both patients and staff, thereby inferring additional benefits over NPS (such as reduced staff training). Ultimately, healthcare organizations may need to take sufficient time to assess the suitability of NPS to their organization, and ensure adequate resourcing and training for successful implementation, data management and ongoing utilization for service improvement.

These findings may have significant implications for healthcare policy, particularly as NPS has been used as a mandatory measure for patient experience (with FFT in NHS), with the results used to monitor and benchmark healthcare system performance.^33^ While the use of NPS may be valuable for some purposes, this requires integration with more in‐depth measures at a policy and system level, which will assist feedback to be used as a driver for service improvement. For future policy development, further consideration may be necessary before mandating the use of NPS as a sole and primary measure of patient experience, particularly in consideration of the changes made with the FFT. Future research could include multisite studies with rigorous study design, which would support the development of evidence‐based utilization of patient experience measures. Furthermore, NPS is used across the world; however, this review highlights that there are cultural differences to consider when implementing NPS.^16^, ^19^ As such, future research may include an evaluation of NPS in multiple countries, with an assessment of cultural suitability and consideration of the surveying modifications that may be required to appropriately adapt NPS for different countries (such as the scoring for NPS subcategories). This would help to ensure that, if NPS is used, then it is appropriate and suitable to the context in which it is being implemented.

### 4.2 Strengths and limitations

This review provides a useful synthesis of evidence for many healthcare organizations that are currently using NPS or are considering implementing NPS. The use of a systematic review methodology of peer‐reviewed literature strengthens this review. Yet, there are review limitations, including the exclusion of grey literature (such as case studies and commentaries). This occurred so that the review could focus on the evaluation of peer‐reviewed research; however, it is acknowledged that this may have led to the omission of useful information in the review. Patient experience experts and information specialists were consulted during the development of this study to support a comprehensive understanding and inclusion of the appropriate keywords and search terms. However, it is recognized that NPS has been incorporated into various healthcare settings with modified survey names (e.g., ‘Friends and Family Test’). Therefore, due to the lack of standardized terminology, the ability of this search strategy to include all relevant articles may be limited. In addition, this review had limitations with the literature search conducted in five databases and including only articles written in English, which may have overlooked information from unpublished studies, studies written in other languages and studies published in journals not linked to the searched databases. Furthermore, 10 out of 12 studies were conducted in the United Kingdom, which could produce contextual bias, and several of the included studies used opportunistic sampling relating to the implementation of the FFT. As such, it is suggested that further research could be conducted to validate NPS surveying, using rigorous research methodology, and is performed in a variety of countries and different healthcare settings.

## 5 CONCLUSION

NPS is increasingly being implemented to evaluate patient experience, despite a lack of peer‐reviewed research to support its use in the healthcare setting. NPS may be an appealing option for healthcare organizations as a quick and simple measurement that can generate a large number of responses; however, the literature suggests that this data does not necessarily translate to healthcare improvement. As the foundation of NPS is based upon willingness to recommend, NPS appears best‐suited to healthcare contexts that allow for the selection of treatment, such as primary care and elective surgery. Although promoted as the ‘Ultimate Question’, research suggests that NPS may be insufficient as a stand‐alone metric. Instead, NPS may be more effectively used as part of a more comprehensive feedback process, such as multi‐item surveying. Research does not support the use of NPS for widespread benchmarking, hence NPS may be more suited to assess localized performance. Overall, there are limitations to the use of NPS, so healthcare organizations may need to consider a range of factors before selecting NPS as a primary measure of patient experience. Further research with rigorous methods is needed to validate the use of NPS specifically for healthcare settings.

## CONFLICT OF INTEREST

The authors declare no conflict of interest.

## Data Availability

The authors confirm that the data supporting the findings of this study are available within the article and its Supporting Information Materials.
